# A scaffolded and annotated reference genome of giant kelp (*Macrocystis pyrifera*)

**DOI:** 10.1186/s12864-023-09658-x

**Published:** 2023-09-13

**Authors:** Jose Diesel, Gary Molano, Gabriel J. Montecinos, Kelly DeWeese, Sara Calhoun, Alan Kuo, Anna Lipzen, Asaf Salamov, Igor V. Grigoriev, Daniel C. Reed, Robert J. Miller, Sergey V. Nuzhdin, Filipe Alberto

**Affiliations:** 1 Department of Molecular and Computational Biology, University of Southern California, Los Angeles, CA USA; 2 Department of Biological Sciences, University of Wisconsin-Milwaukee, Milwaukee, WI USA; 3 U.S. Department of Energy Joint Genome Institute, Lawrence Berkeley National Laboratory, Berkeley, CA USA; 4 Department of Plant and Microbial Biology, University of California Berkeley, Berkeley, CA USA; 5 Marine Science Institute, University of California at Santa Barbara, Santa Barbara, CA 93106 USA

**Keywords:** Genome, Giant kelp, *Macrocystis pyrifera*, Brown macroalga, Population genetics

## Abstract

*Macrocystis pyrifera* (giant kelp), is a brown macroalga of great ecological importance as a primary producer and structure-forming foundational species that provides habitat for hundreds of species. It has many commercial uses (e.g. source of alginate, fertilizer, cosmetics, feedstock). One of the limitations to exploiting giant kelp’s economic potential and assisting in giant kelp conservation efforts is a lack of genomic tools like a high quality, contiguous reference genome with accurate gene annotations. Reference genomes attempt to capture the complete genomic sequence of an individual or species, and importantly provide a universal structure for comparison across a multitude of genetic experiments, both within and between species. We assembled the giant kelp genome of a haploid female gametophyte de novo using PacBio reads, then ordered contigs into chromosome level scaffolds using Hi-C. We found the giant kelp genome to be 537 MB, with a total of 35 scaffolds and 188 contigs. The assembly N50 is 13,669,674 with GC content of 50.37%. We assessed the genome completeness using BUSCO, and found giant kelp contained 94% of the BUSCO genes from the stramenopile clade. Annotation of the giant kelp genome revealed 25,919 genes. Additionally, we present genetic variation data based on 48 diploid giant kelp sporophytes from three different Southern California populations that confirms the population structure found in other studies of these populations. This work resulted in a high-quality giant kelp genome that greatly increases the genetic knowledge of this ecologically and economically vital species.

**Supplementary Information:**

The online version contains supplementary material available at 10.1186/s12864-023-09658-x.

## Introduction

*Macrocystis pyrifera* (giant kelp) is the world’s largest macroalga and one of the fastest growing multicellular autotrophs on Earth, increasing in mass by an average of 3.5% per day in favorable locations [1]. It forms extensive subtidal forests on shallow reefs in temperate seas that are among the most productive ecosystems on Earth [2, 3]. Importantly, the high primary production and three-dimensional structure of giant kelp forests provide habitat for hundreds of species, ranging from microscopic invertebrates to different types of fish and mammals [4]. It’s not surprising that Darwin considered giant kelp forests analogous to terrestrial rainforests, owing to the impressive species diversity sustained in both habitats [5].

As a haplodiplontic organism, giant kelp alternates between a macroscopic diploid sporophyte stage and a microscopic haploid gametophyte stage. The diploid sporophyte releases haploid zoospores into the water column that eventually settle on the ocean floor and develop into sexed gametophytes. Female gametophytes release a pheromone that triggers the release and attraction of sperm from the male gametophyte towards their oogonia [6]. After fertilization, young diploid sporophytes develop into the macroscopic adults composing kelp forests [4]. The dispersal distance of the zoospores depends on many factors, including water motion [7–9]. Successful colonization depends on opposite sex zoospores settling with millimeters of each other on suitable habitat [10, 11], which is dependent on the size of nearby kelp forests as well as the number and synchronous timing of zoospores released by them [12–15].

Interest in giant kelp as an aquaculture crop has increased, with the global algal aquaculture market in 2020 producing 35 million tons of algae worth $16.5 billion [16]. Giant kelp is one of the main commercial sources of alginate, a long chain polysaccharide found in the cell walls of brown macroalgae, with uses in food as a thickener and in medicine as a hydrogel [17, 18]. Giant kelp also has a range of other applications including human food, animal feed, cosmetics, pharmaceuticals, and fertilizers [19]. Furthermore, due to its fast growth rate and limited composition of lignin and cellulose, giant kelp has been identified as a potential marine crop for biofuel [20]. However, the large-scale cultivation of giant kelp lags that of other kelps such as *Saccharina japonica* and *Undaria pinnatifida*, which are grown in China, Korea, and Japan as human food sources and have undergone selective breeding programs since the 1950s [21–23].

Genomics can greatly benefit aquaculture production by assisting in breeding efforts to increase crop productivity, increase the quality of specific compounds in the crop, and increase resistance to stress, disease, and bacterial infection [24]. The first brown macroalga to have its genome completely sequenced was *Ectocarpus siliculosus*, which has served as a model species [25]. Both *Saccharina japonica* and *Undaria pinnatifida* have since had their genomes sequenced, an important step in breeding programs because reference genomes provide an individual’s complete genetic information that can be universally compared across experiments [26–28].

Previous research has identified the need for improved cultivars of brown macroalgae and this improvement can be expedited in giant kelp by increasing the availability of genomic tools, such as a quality reference genome and sequencing experiments [29, 30]. However, the available genomic references for giant kelp are thus far limited to a giant kelp transcriptome in 2013, a heavily fragmented genome with an estimated completeness of only 10% based on stramenopile single copy orthologs, and a set of gene models derived from reciprocal blasts against *Ectocarpus siliculosus* [31–33]. Prior research identified a northern hemisphere origin for giant kelp based on phylogenetic analysis of the ribosomal internal transcribed regions [34]. Further molecular dating in conjunction with fossil records estimate that giant kelp emerged as a species ~6 million years ago and initially was found in the colder waters in the Pacific Ocean off the Alaskan coast [35]. The global hotspot of microsatellite genetic diversity for giant kelp is presently in the Southern California Bight in the northeast Pacific, reflecting a Pleistocene glacial refugia for the species [36]. For example, a draft of the presented annotated genome was used to examine the genomic differences between two different morphologies of giant kelp in the Northern and Southern hemispheres [37]. Therefore, in our efforts to support giant kelp domestication,, we improved giant kelp’s genomic resources by assembling a nuclear genome with 92% of sequences scaffolded to chromosomal levels, and investigated three Southern California populations for markers that can be used in future selective breeding models for giant kelp aquaculture.

## Results

### Genome sequencing, assembly, and annotation

**Fig. 1:**
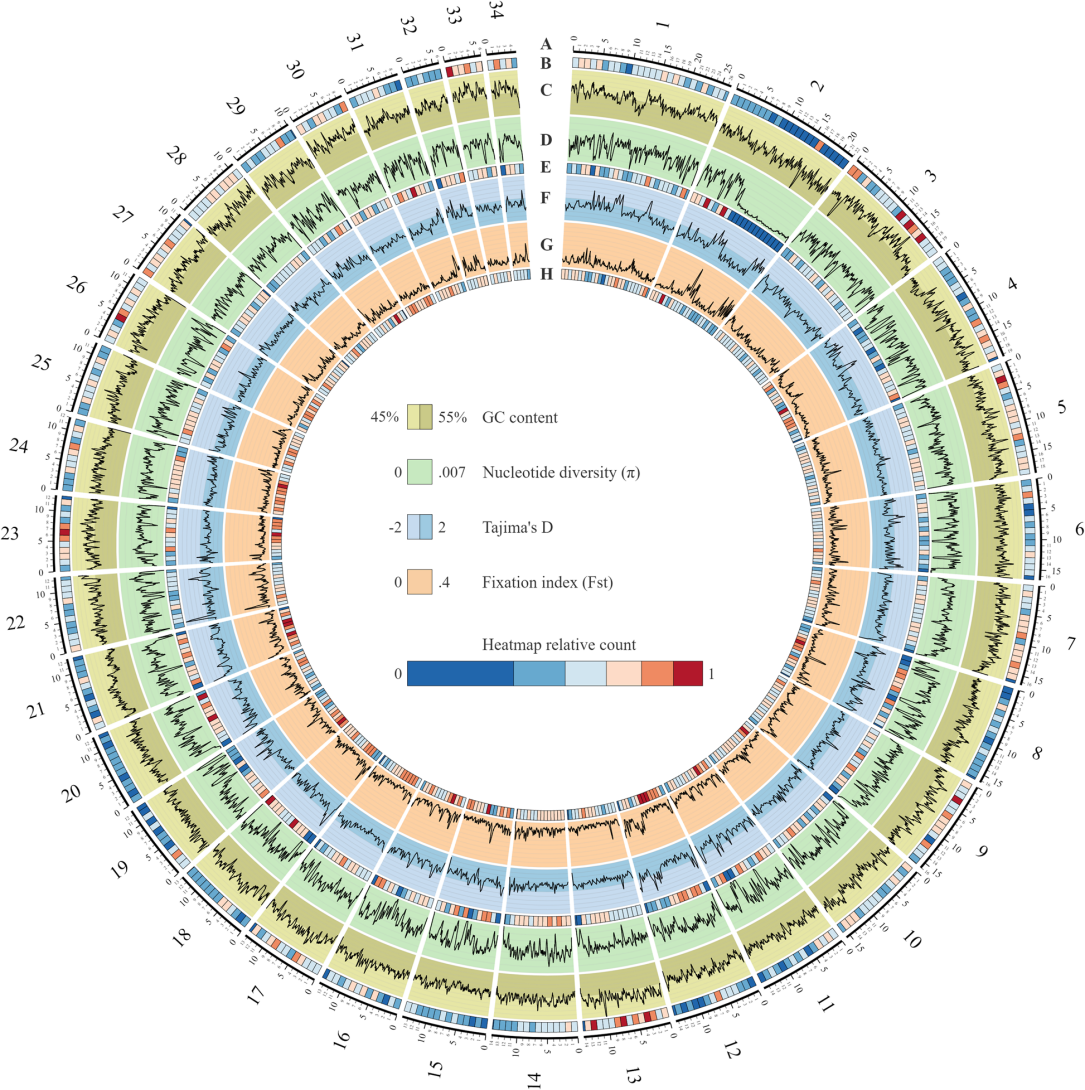
Circos plot of first 34 contigs of the giant kelp genome. Different aspects of the genome are represented in each concentric circle. **A** Scaffold size in MB. **B** Gene density heatmap. **C** Percentage of GC ranging from 45 to 55%. **D** Nucleotide diversity ranging from 0 to 0.007. **E** SNP density heatmap. **F** Tajima’s D values ranging from -2 to 2. **G** Fst values ranging from 0 to 0.4. **H** TE density heatmap. Line values are plotted on the same 200 kb sliding window with 40 Kb intervals while heatmaps are plotted over the same 1 MB windows. Heatmaps are in a 1.75 log scale for greater dynamic range at higher values

We extracted DNA from a single female haploid gametophyte and sequenced the DNA using PacBio Sequel II technology obtaining 57 GB of long reads with average read length of 15.8 kb, representing a coverage of approximately 100 × of our estimated 513–542 MB giant kelp’s genome. Our de novo assembly generated 1,033 contigs with a total length of 540 MB and N50 of 1.7 MB. After decontamination, scaffolding using Hi-C technology was performed by Phase Genomics and clustered 96.82% of the contigs into 35 clusters, resulting in a final assembly of 35 scaffolds and 188 contigs with a total genome size of 537 MB and N50 of 13.6 MB (Table 1). Scaffolds average 13.3 MB and 34 out of 35 are larger than 4 MB (Fig. 1).

**Table 1:** Genome statistics comparison between the genomes of *Macrocystis pyrifera* (assembled in this study), *Ectocarpus sp.* [25], *Saccharina japonica* [26], and *Undaria pinnatifida* [27]

	***Macrocystis pyrifera***	***Ectocarpus siliculosus***	***Saccharina japonica***	***Undaria pinnatifida***
**Length**	537Mb	196Mb	548Mb	511Mb
**# Contigs**	223	30	4598	114
**Largest Contig**	26Mb	19Mb	2.3Mb	32Mb
**N50**	13.6Mb	6.5Mb	0.34Mb	16.5Mb
**# of N per 100Kb**	12.99	2601.51	1541.53	49.29
**# Genes**	25,919	16,271	18,732	14,300
**% GC content**	50.37	53.59	49.35	50.14

**Fig. 2:**
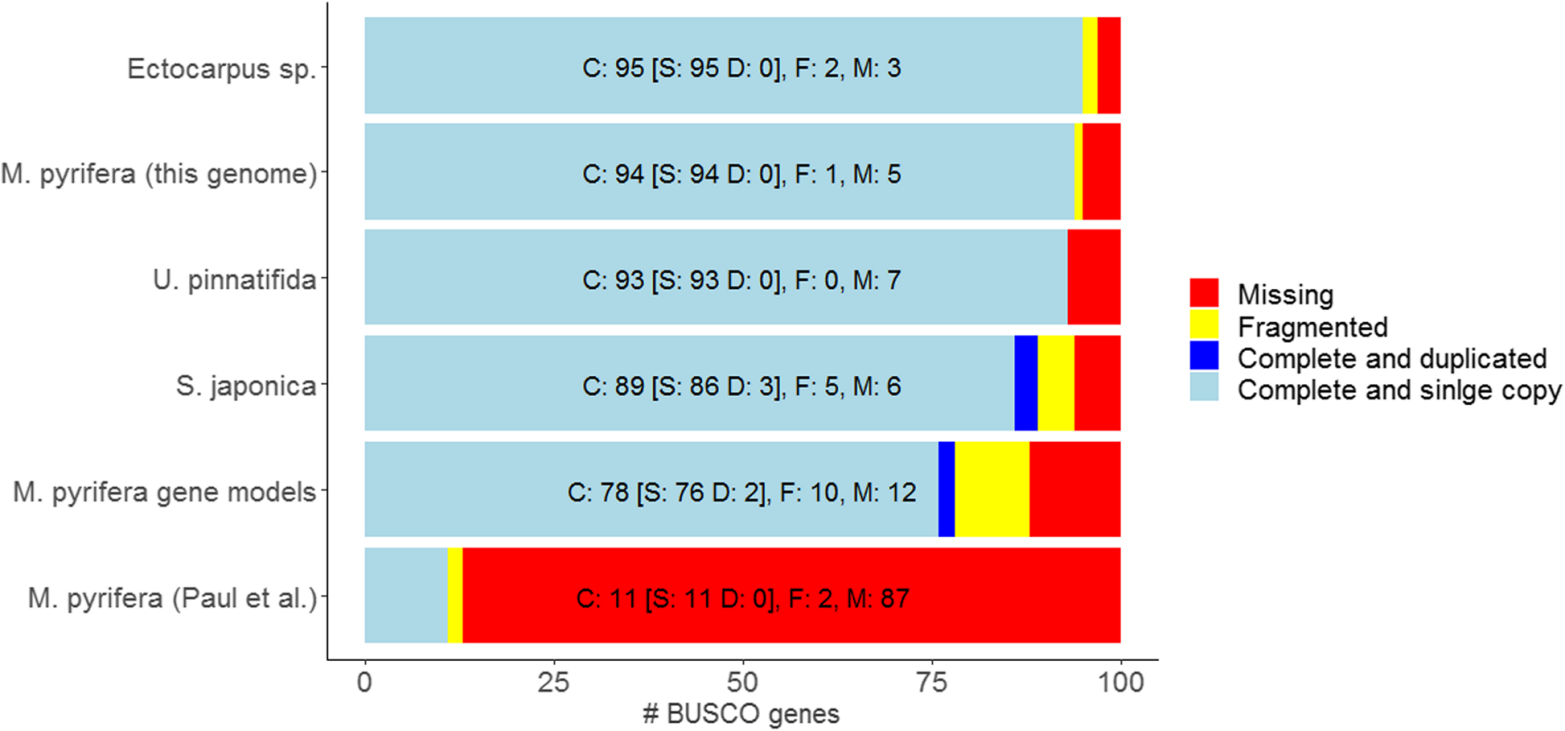
Comparison of BUSCO assessment of genome completeness based on the stramenopiles_odb10 dataset between the macroalgaes genomes of *Macrocystis pyrifera* (assembled in this study), *Ectocarpus sp.* [25], *Saccharina japonica* [26], *Undaria pinnatifida* [27], *Macrocystis pyrifera* gene models from Molano et al. 2022 [34], and genome from Paul et al. 2022 [33]

We used BUSCO for an assessment of gene content completeness of our genome, as it looks for single copy conserved orthologs in a given taxonomic group. The stramenopile BUSCO analysis showed that the giant kelp genome (*Macrocystis pyrifera*) compared favorably to other published brown macroalgae genomes, with 94 complete BUSCO genes, 1 fragmented gene, and 5 missing genes. We compared the assembled giant kelp genome presented here with other existing giant kelp resources, highlighting the improvements this new assembly offers. This includes a recently published, highly fragmented genome that merely encompass 11 completed stramenopile BUSCO genes, as well as a limited set of gene models that underwent reciprocal blast against *Ectocarpus siliculosus* and encompassed 78 complete BUSCO genes (Fig. 2) [32, 33].

We report the genome assembly statistics of our giant kelp genome and the three major brown macroalgae genomes, *Ectocarpus siliculosus, Saccharina japonica,* and *Undaria pinnatifida*. Our N50 for giant kelp is 13,669,674, which is greater than the N50 for *Ectocarpus siliculosus* and *Saccharina japonica*, but less than that of *Undaria pinnatifida*. Our giant kelp genome also contained fewer missing bases per 100,000 bases out of the other brown macroalgae genomes. The giant kelp estimated genome size based on kmer frequency from the Pacbio corrected reads is 513 MB for k = 25 and 542 MB for k = 31 (Supplementary Fig. 1). Our assembled genome is 537 MB, which falls within this predicted range of genome sizes. The 537 MB genome size is almost half of the upwards estimate from flow cytometry and microspectrophotometry of 686 MB to 1176 MB, which may have been inflated due to the polytenic state of the female gametophytes of giant kelp [38, 39].

Protein-coding genes were predicted using ab initio, homology-based, and transcriptome-based modelers, which after filtering resulted in the prediction of 25, 919 protein coding genes, greater than predictions of *Saccharina japonica* and *Ectocarpus siliculosus*, with 18,733 and 16,256 respectively (Table 1 and Fig. 1B) [25, 26]. Brown macroalgae have a UV sex determining system in which sex determining regions (SDR) determine the sex of an individual gametophyte. SDR typically are less gene dense, contain large repeats of DNA, do not recombine and thus can expand in size as the SDR accumulates repetitive DNA [40]. Scaffold 2 contains fewer genes than other scaffolds of similar size (Scaffold 1 contains 1135 genes, Scaffold 2 600 genes and Scaffold 3 1194 genes). In the second half of scaffold 2, the gene density and the pairwise genetic diversity decreases in comparison with the rest of the genome (Fig. 1B). If Scaffold 2 contains the SDR for giant kelp, it should only contain female markers for kelp SDR regions. After blasting the kelp genome using previously identified male and female SDR markers, the second half of scaffold 2 contained the four sex determining markers specific for females in giant kelp [41]. This fits expectation as a female gametophyte was the individual sequenced in this experiment. The presence of the female sex determining markers in combination with decreases in gene density and genetic diversity indicates that scaffold 2 contains the putative SDR region of giant kelp.

### Genome comparative analysis

**Fig. 3:**
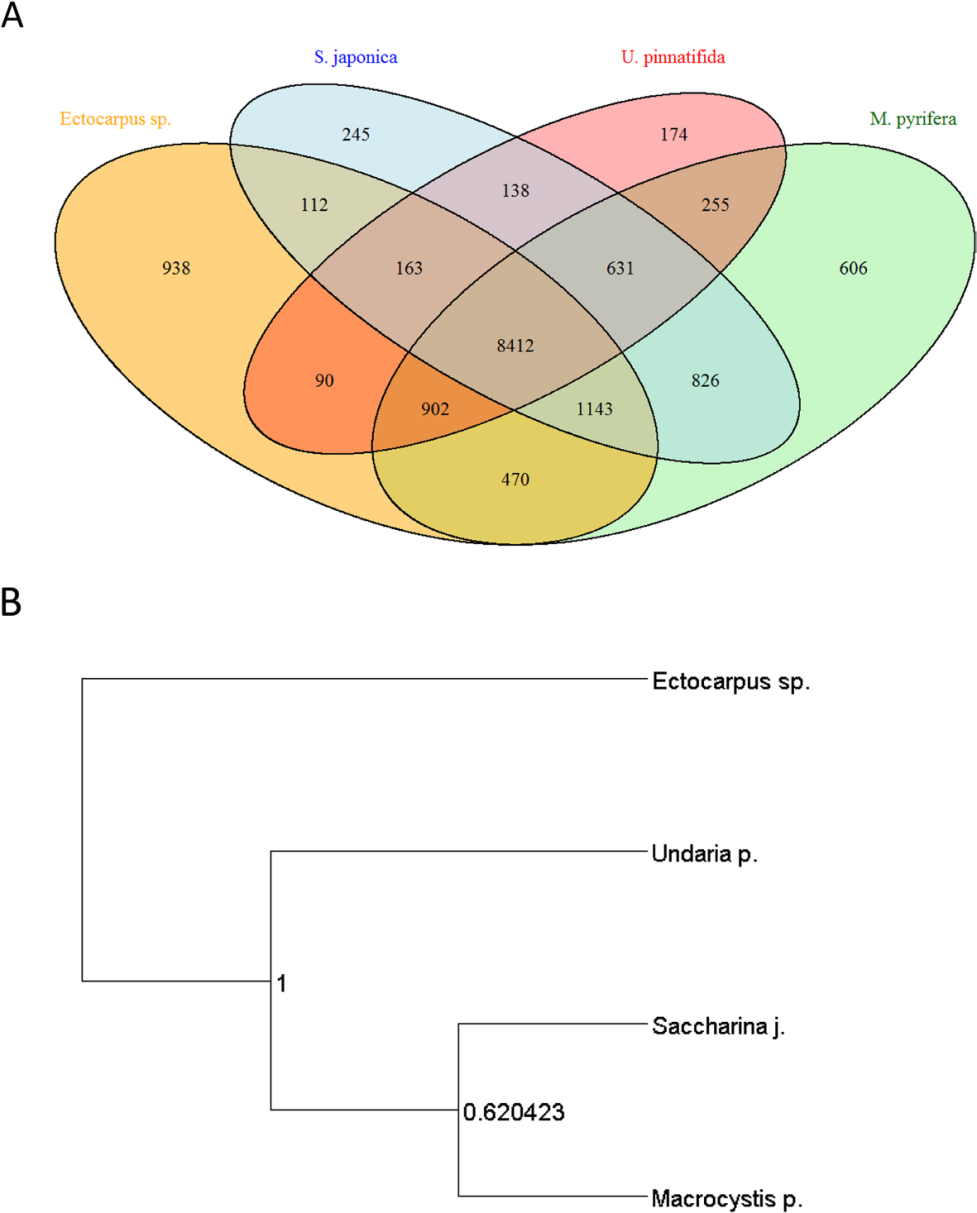
Analysis of orthologs. **A** Protein comparative analysis of orthologs between giant kelp and three other relevant macroalgal species (*Ectocarpus sp.*, *Saccharina japonica* and *Undaria pinnatifida*) using Orthofinder. Numbers represent shared orthogroups between species. **B** Species tree inferred by OrthoFinder

To identify orthologous genes among other relevant macroalgae, we performed a comparative analysis using Orthofinder between *Macrocystis pyrifera*, *Ectocarpus siliculosus*, *Saccharina japonica* and *Undaria pinnatifida*. A total of 70,317 genes were analyzed, of which 61,267 (87.1%) were assigned to a total of 14,001 orthogroups; of those, 7,660 were present in all four species (Fig. 3A and B).

Differences in genome size between Ectocarpales and Laminariales have been explained by the expansion of repetitive elements in the larger genomes of Laminariales [42]. Following this pattern, we found repetitive sequences to total 57.6% (309 Mb) (Fig. 1H and Supplementary Table 1) of giant kelp’s genome, similar to what was found in the laminarian *U. pinnatifida* (52.1%) and contrasting with the lower amount of repetitive sequences in *E. siliculosus* (22.7%).

**Fig. 4:**
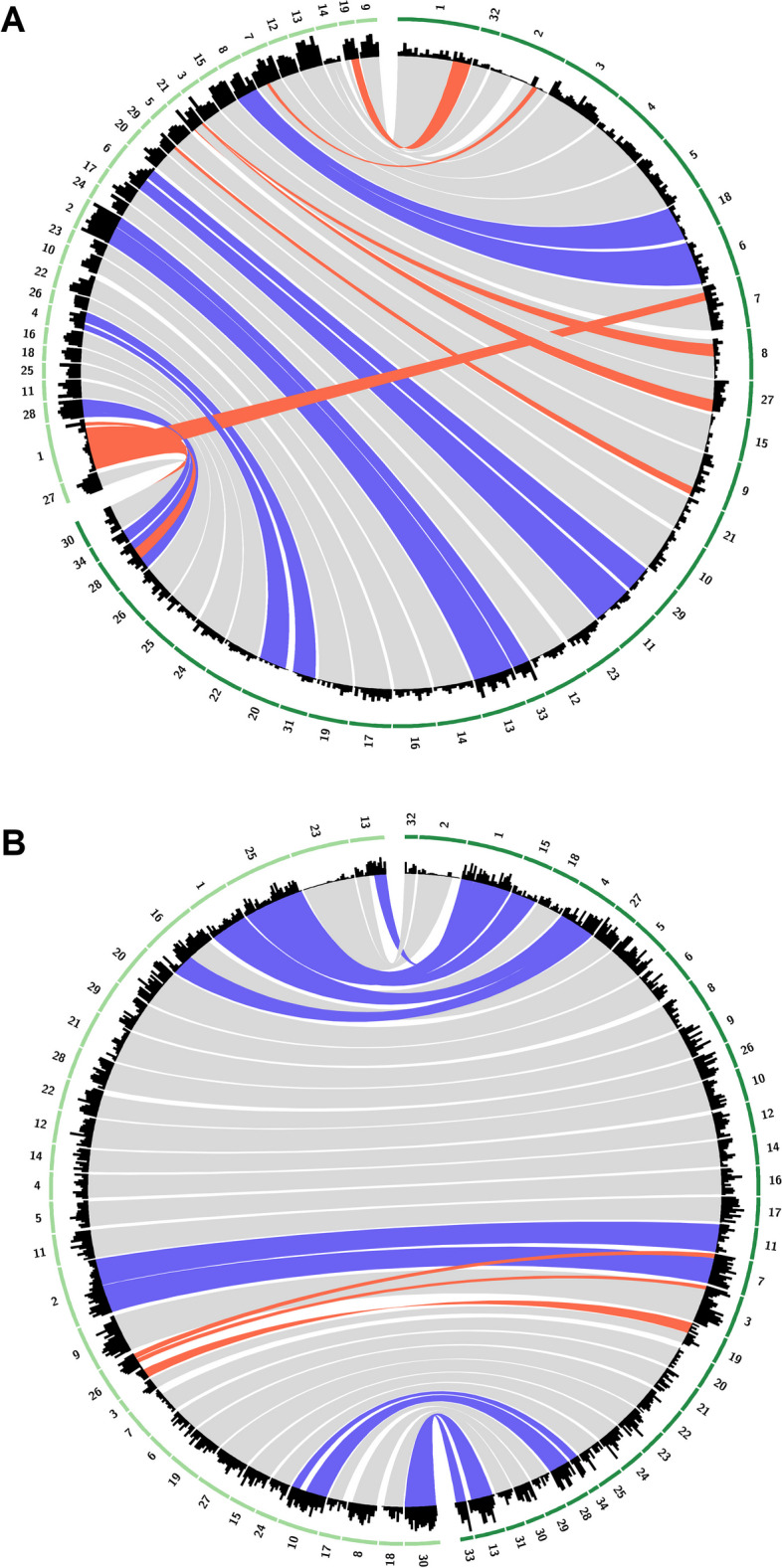
Synteny between the *Macrocystis pyrifera* genome (dark green) and the genomes of **A** *Ectocarpus siliculosus* and **B** *Undaria pinnatifida* (light green). Bands represent clusters of at least 10 single copy orthologs no more than 3 MB apart. Purple bands are potential chromosome splitting or fusion. Gray bands represent scaffolds that share the highest number of orthologs and, therefore, are most syntenic. Red bands represent orthologs in different scaffolds. When multiple bands overlap, bands with fewer number of orthologs superimpose bands with higher numbers of orthologs for easier visualization. Histogram represents density of single copy orthologs in 1 MB windows

Overall synteny is conserved between *E. siliculosus* and *M. pyrifera* single copy orthologs, but there are signs of chromosomal rearrangement including the splitting of four chromosomes and fusion of two, which explains the chromosome number difference between the two species (Fig. 4A). As expected, synteny is more conserved between *M. pyrifera* and *U. pinnatifida*, both in the order Laminariales, with signals of chromosomal splitting with similar gene density but less chromosomal rearrangement (Fig. 4B).

**Fig. 5:**
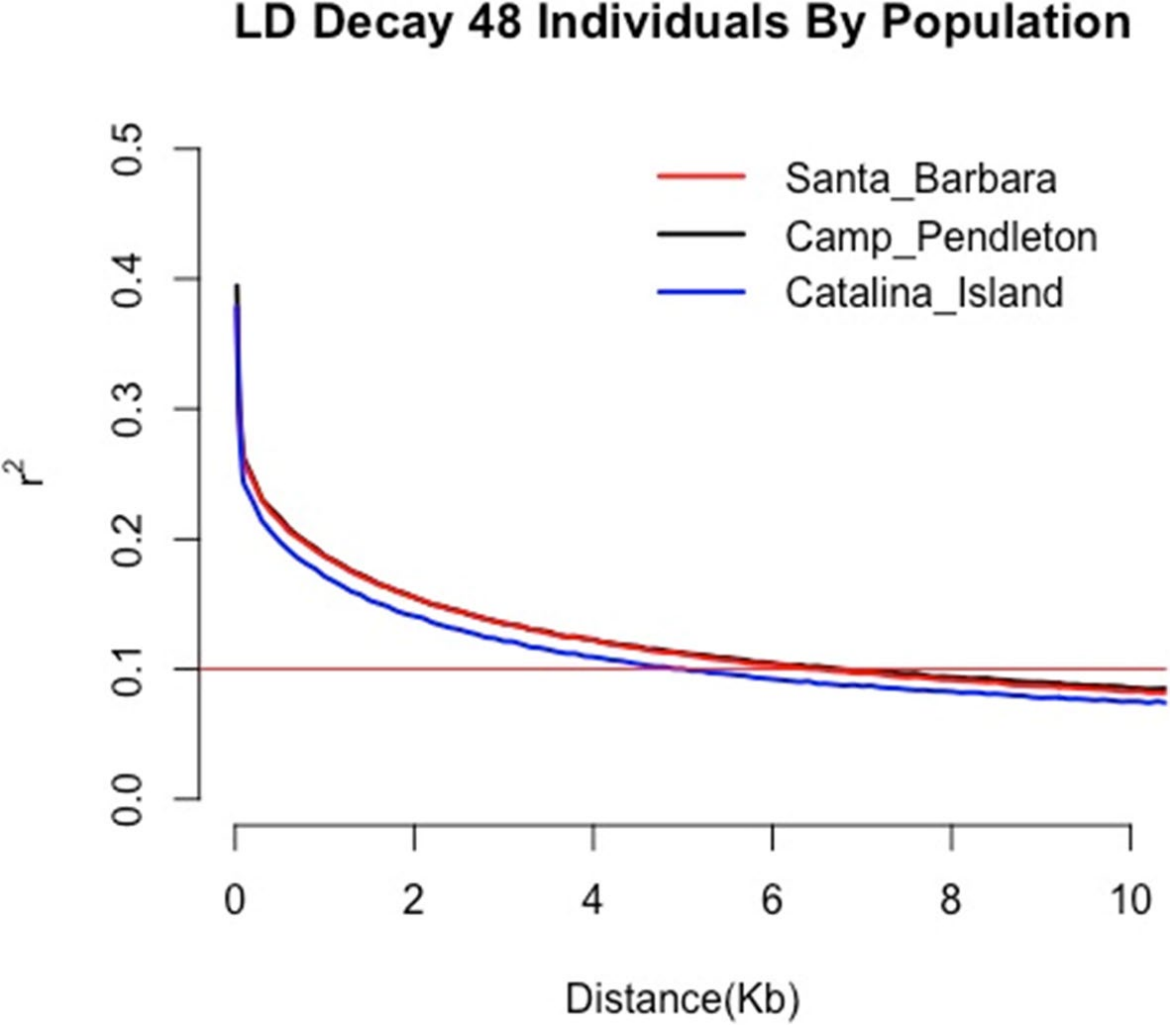
LD decay curve for each sampling population separately

We also calculated linkage disequilibrium for the 48 samples for each population, as it can be used to improve selective breeding models. Using r^2 = 0.1 as the LD threshold we estimated the LD block size to be ~5.5 kb for the Catalina Island population and ~ 6 kb for the Camp Pendleton and Santa Barbara populations (Fig. 5). Pairwise genetic diversity *(π*) across the whole giant kelp genome estimated *π* = 0.0035 (Fig. 1C). Tajima’s D was overall negative throughout the genome, with an average of -1.17522 across 200 kilobase windows (Fig. 1F). Variant effect annotation showed that 2.66% of the variants were found inside exons with 53.1% predicted to be missense, while intergenic variants accounted for 32.73% of all variants (Supplementary Table 2 and Supplementary Fig. 2).

## Discussion

Our study presents an improved annotated and scaffolded giant kelp reference genome capable of supporting a genomics approach for the ongoing domestication and conservation efforts for this species. This giant kelp reference genome compares favorably to the three published major brown macroalgae genomes, *Ectocarpus siliculosus*, *Saccharina japonica*, and *Undaria pinnatifida*, with similar N50 values (genome contiguity) and BUSCO scores (genome completeness) [25–27, 43]. Additionally, our study presents a comprehensive analysis of whole genome linkage disequilibrium, nucleotide diversity and Tajima’s D. Compared to previous giant kelp genomes, the giant kelp assembly presented here vastly improves on genome contiguity and completeness, in particular when comparing BUSCO scores (94% compared to 11%) [32]. Therefore, we anticipate that the scaffolded giant kelp genome presented here will be the universal reference for future giant kelp genomic projects.

## Conclusion

The giant kelp genome presented in this study will assist in ongoing giant kelp domestication and conservation efforts by providing a reference genome that can be used as a comparative benchmark between giant kelp individuals sequenced in other kelp genetic studies. The functional annotations of the genome can help pinpoint the genomic locations of genes of interest for domestication for further genetic variation analysis. The use of a conserved gene model data set for phylogenetic studies may be sufficient in giant kelp as the strong population structure seen in the diploid data concurred with the results from the gene models.

## Materials and methods

### Data collection and sequencing

Sporophylls (spore-producing blades containing sporangia in diploid sporophytes) were collected from giant kelp individuals attached to the rocky substrate near Catalina Island using SCUBA. Sporophylls were then sent to the University of Wisconsin-Milwaukee, where spores were released into Instant Ocean water. After 15–20 days, single gametophytes were isolated into single genotype cultures. To produce sufficient biomass required to extract 2 μg of high quality and high molecular weight genomic DNA, we cultivated a single haploid female gametophyte from the Catalina Island population (CI_03). To avoid gametogenesis, we grew the culture vegetatively in red light (30 μmol photons m^−2^ s^−1^), at 12ºC temperature and 12:12 h (day:night) photoperiod.

To ensure the least amount of contamination during PacBio sequencing, the culture was repeatedly treated with antibiotics until no bacterial colonies would form when plated [44, 45]. High molecular weight DNA was extracted using the protocol of Doyle and Doyle (1987) with minor modifications. Essentially, young gametophytes that had been flash frozen and kept frozen at -80C were ground to a fine powder in a frozen mortar with liquid N_2_, followed by very gentle extraction in CTAB buffer (that included proteinase K, PVP-40 and beta-mercaptoethanol [46]) for 20 min at 37 °C and 20 min at 50 °C. After centrifugation, the supernatant was gently extracted twice with 24:1 chloroform: iso-amyl alcohol. The upper phase was adjusted to 1/10^th^ volume with 3 M Sodium acetate (pH = 5.2), gently mixed, and DNA precipitated with iso-propanol. DNA was collected by centrifugation, washed with 70% Etoh, air dried for a few minutes, and dissolved thoroughly in 1 × TE at room temperature. Size was validated by pulsed field electrophoresis.

Sequencing of sheared DNA > 30 kb was performed at the Arizona Genome Institute on a Pacbio Sequel II Platform. A SMRTbell Express Template Prep Kit 2.0 was used for library preparation and a Sequel II Binding Kit was used for the sequencing that generated 56 GB of long read data.

### Genome assembly

Contamination from sample collection and library preparation has been found in many different reference genomes, and can be the cause for erroneous results in downstream analysis [47]. Contamination has been identified as a concern in genomes of other brown macroalgae, including *Saccharina japonica* [48]. In order to assemble our genomes using sequences free of most contamination, we loosely aligned our PacBio reads to three different brown macroalgae genomes (*Ectocarpus siliculosus*, *Saccharina japonica* and *Cadosiphon okamuranus*) using Minimap2 map-pb option [49] and excluded from our assembly sequences that did not map to any of the brown macroalgae genomes using Samtools v1.15.1 [50]. We assembled the remaining reads using Canu 1.9 [51] on standard settings resulting in a preliminary assembly of 1,039 contigs containing 539Mbp, which was then polished using Racon v1.5.0 [52].

Because using published genomes to do an initial contamination filter relies on those genomes being contamination free, we analyzed the potential contamination of each contig separately. We split the assembly into individual contigs using faSplit [53] and used diamond blast v2.10 to blast each contig against the Uniprot reference proteome database with an evalue of 1e-15 [54, 55]. We then added the results to the blobtools pipeline using the add --hits command and filtered contigs based on length (contig > 10,000 base pairs), GC content (between 0.35–0.65), coverage (5-300X), and blast classification, keeping contigs identified as phaeophyceae and no-hit. The first round of filtering removed 161 contigs and ~42 MB of sequence. The 870 contigs were then sent to Phase Genomics for scaffolding.

Chromatin conformation capture data was generated using a Phase Genomics (Seattle, WA) Proximo Hi-C 2.0 Kit, which is a commercially available version of the Hi-C protocol [56]. Following the manufacturer’s instructions for the kit, intact cells were crosslinked using a formaldehyde solution, digested using the DPNII restriction enzyme, end repaired with biotinylated nucleotides, and proximity ligated to create chimeric molecules composed of fragments from different regions of the genome that were physically proximal in vivo, but not necessarily genomically proximal. Continuing with the manufacturer’s protocol, molecules were pulled down with streptavidin beads and processed into an Illumina-compatible sequencing library. Sequencing was performed on an Illumina NovaSeq.

Reads were aligned to the 870 contig draft assembly giant kelp genome also following the manufacturer’s recommendations (https://phasegenomics.github.io/2019/09/19/hic-alignment-and-qc.html). Briefly, reads were aligned using BWA-MEM with the -5SP and -t 8 options specified, and all other options default [57]. SAMBLASTER was used to flag PCR duplicates, which were later excluded from analysis [58]. Alignments were then filtered with samtools using the -F 2304 filtering flag to remove non-primary and secondary alignments [50]. Putative misjoined contigs were broken using Juicebox based on the Hi-C alignments [59, 60].

Phase Genomics’ Proximo Hi-C genome scaffolding platform was used to create chromosome-scale scaffolds from the corrected assembly as described in Bickhart et al. [61]. As in the LACHESIS method, this process computes a contact frequency matrix from the aligned Hi-C read pairs, normalized by the number of restriction sites on each contig, and constructs scaffolds in such a way as to optimize expected contact frequency and other statistical patterns in Hi-C data [62]. Approximately 20,000 separate Proximo runs were performed to optimize the number of scaffolds and scaffold construction in order to make the scaffolds as concordant with the observed Hi-C data as possible. Finally, Juicebox was again used to correct scaffolding errors.

After scaffolding and preliminary annotation, we found several contigs that had been discarded from the assembly contained stramenopile_odb10 BUSCO genes. We determined that our initial filtering with the blobtools pipeline had been too strict, mostly due to misclassification of the contigs during the blast analysis. The misclassification of sequences has been shown to be an increasing problem in sequence databases [63, 64]. After manually checking the 161 discarded contigs for potential candidate giant kelp contigs, we added 29 more contigs into the giant kelp assembly, increasing the size of the genome ~38 MB to 537 MB (Supplementary Fig. 3). The additional contigs also raised the BUSCO score using stramenopile_odb10. Unfortunately, the initial 870 contigs had been already scaffolded, and completely re-scaffolding the genome was cost prohibitive.

### Genome completeness

To assess genome completeness using single copy orthologs, we used BUSCO v5.2.1 in genome mode and in conjunction with the stramenopile_odb10 dataset to compare our giant kelp genome to the publicly available genomes of *Ectocarpus siliculosus, Saccharina japonica,* and *Undaria pinnatifida* [64]. BUSCO (Benchmarking Universal Single Copy Orthologs) searches a genome for single copy ortholog proteins that are in data sets of specific lineages. BUSCO scores from the giant kelp genome can then be compared against other brown macroalgal genomes, with the higher number of BUSCO genes showing a more complete genome and lower number of duplicated genes showing less duplication artifacts from assembly.

Other methods to check genome completeness include genome contiguity, usually measured using the N50 statistic, and comparing genome size to estimated genome size [65]. N50 is the length of the shortest contig or scaffold for which contigs or scaffolds with greater or equal length cover at least 50*%* of the assembly [66]. Here we compared the same genomes using QUAST v5.0.2 with standard settings in order to generate genome assembly statistics [43].

When assessed using microspectrophotometry, the potential genome sizes of giant kelp gametophytes ranged from 882 MB to 1,176 MB [67]. However, when using flow cytometry, the giant kelp genome was estimated to be 686 MB [68]. This discrepancy may be explained by heterogeneous amounts of nuclear material in giant kelp gametophytes. Giant kelp female gametophytes have been shown to sometimes have double the genetic material compared to most male gametophytes [39]. Other brown macroalgae, such as *Saccharina latissima*, also have variable amounts of DNA content in their haploid tissue [38]. Therefore, using physical parameters to accurately calculate the genome size of brown macroalgae may require homogeneous mixes of cells with the same levels of DNA content. Computational methods can estimate the genome size of an organism based on approximating the repeat structure of sequenced shotgun reads from a genome [69]. Sometimes, the genome estimates using kmers, or unique subsequences of DNA of length k, may produce different lengths of the genome compared with physical measurements from flow cytometry [70]. Since the physical estimates of the giant kelp genome are not consistent, we compared them against a kmer based estimate. Long reads from Pacbio sequencing have been shown to accurately estimate genome size using kmers as long as the reads have been corrected [71, 72]. To computationally estimate genome size, we used the corrected Pacbio reads generated by Canu with specific settings for Sequel II reads: correctedErrorRate = 0.035 utgOvlErrorRate = 0.065 trimReadsCoverage = 2 trimReadsOverlap = 500 [51]. We then counted the number of kmers found in the corrected reads using the kmercounter program kmc and two different kmer sizes, with k = 25 and k = 31 [71]. Kmer distributions of both k = 25 and k = 31 was plotted, and genome size estimate was done by summing the total number of kmers and dividing by the mean coverage of kmers in the genome.

### Annotation

The giant kelp nuclear genome assembly was annotated using the JGI Annotation pipeline [72, 73]. The following steps describe the pipeline in brief. The genome assembly was masked for repeats using RepeatMasker [74] with the RepBase library [75] and the most frequent repeats (more than 150 copies) identified by RepeatScout [76]. Protein-coding gene models were predicted using the following gene modelers: ab initio modelers Fgenesh [77] and GeneMark [78], homology-based Fgenesh+ and GeneWise [79] seeded by BLASTx alignments against the NCBI NR database, and transcriptome- based modelers Fgenesh, combest [80], and Braker [81]. For use in gene prediction, transcriptome assemblies were generated from Illumina RNAseq reads (Accession numbers: SRR5026366, SRR5026588, SRR5026590, SRR5026591, SRR5026593, SRR5026594, SRR3544557, SRR3615022) using Trinity (v2.11.0) [82], and as input to Braker, RNA reads were mapped to the genome using HISAT2 [83]. To select the best representative gene model at each locus, automated filtering was performed based on homology and transcriptome support. In addition, genes with similarity to transportable elements (TE), containing known TE-related Pfam domains, or lie within repeat-masked regions were excluded from the annotated gene set. Finally, the protein sequences of the predicted gene models were functionally annotated using SignalP v3 for signal sequences [84], TMHMM for transmembrane domains [85], InterproScan for protein domains [86], and homologs based on Blastp alignments against the NCBI NR, SwissProt, and KEGG [87] databases.

Annotation of transposable elements was done using RepeatModeler v2.0.3 with LTRStruct option and genomeSampleSizeMax of 81 Mb [88]. The output was classified using RepeatMasker v4.1.2 on standard settings [74]. Genome assembly and annotations are available from the JGI algal genome portal PhycoCosm (https://phycocosm.jgi.doe.gov/Macpyr2) [89].

### Comparative analysis

The protein datasets from *Macrocystis pyrifera*, *Ectocarpus siliculosus*, *Saccharina japonica*, and *Undaria pinnatifida* were used for ortholog analysis with Orthofinder v2.5.4 and visualization of species tree was done with Dendroscope [90, 91]. The position of single copy orthologs between *M. pyrifera* and *E. siliculosus* were used to determine synteny between the two genomes. Circos v2.30.1 [91] was used to graph links between orthologs in each genome. Clusters where three or more orthologs have no more than 1 MB of distance between them were graphed as bands linking their respective position in each genome.

### SNP calling and population genetics

Raw Illumina reads from 49 giant kelp diploid individuals from three Southern California were downloaded from NCBI (https://www.ncbi.nlm.nih.gov/bioproject/661280). Reads were trimmed of adapter sequences and low-quality tails using Trimgalore [92]. The reads were then aligned to our giant kelp reference genome using Hisat2 v2.1.0 using standard parameters, and the ensuing alignment file was converted to binary and sorted using Samtools v1.9 [50, 83]. Mean depth per individual across the genome calculated using VCFtools v0.1.16 was ~×8, and one individual was removed from the data set due to poor coverage [93]. After removing PCR duplicates, we called variants such as single nucleotide polymorphisms (SNPs) and insertion/deletions (indels), producing a variant call file (VCF) using the GATK4 best practices pipeline [94]. Initial filtering followed the hard filtering suggestions from GATK: “QD < 2.0 || MQ < 40.0 || FS > 60.0 || HaplotypeScore > 13.0 || MQRankSum < -12.5 || ReadPosRankSum < -8.0”. We then filtered the VCF further for population genetics analysis on the following parameters: insertions and deletions removed, biallelic SNPs only, pass quality thresholds of 30, site is called in 90% or more individuals, and each site has a mean depth of 3 reads. Initially, there were 25, 374, 044 SNPs and indels in the raw VCF file before filtering. The filters reduced the number of SNPs to 16,019,851 for downstream analysis.

We performed a principal component analysis (PCA) on the genetic variation using the hard filtered VCF as an input into the SNPrelate v1.22.0. We used the SNPrelate standard pipeline and plotted the PCA using ggplot2 v3.3.2 [95, 96]. We then calculated pairwise genetic diversity and Tajima’s D using VCFtools and the hard filtered VCF file across genomic windows of 200 kb with a step interval of 40 kb, and for each population separately [93]. We also calculated FST between the three populations using VCFtools and the hard filtered VCF file across genomic windows of 200 kb with a step interval of 40 kb.

To analyze linkage disequilibrium and population structure we further filtered the VCF file using vcftools and the commands --maf 0.10 and max-missing 1. This keeps alleles that are present in at least 3 individuals and includes only sites that have no missing data. Phasing of the file was done using py-popgen [97], with beagle package implementation [98]. We then calculated and plotted linkage disequilibrium using PopLDDecay. Prediction on the number of populations was done using faststructure with k values from 1–10 with the built-in chooseK.py script. Different k values were plotted with distruct.py.

### Supplementary Information

**Additional file 1: Supplementary Figure 1.** Kmer distributions generated from corrected Pacbio reads. The genome size can be estimated by summing the total number of kmers found in the Pacbio reads and dividing by the peak of kmers (essentially the coverage for the genome). For a kmer size of k = 25, the coverage was x28 and the genome estimate was ~495 MB. For a kmer size of k = 31, the coverage was x27 and the genome estimate was ~523 MB. **Supplementary Figure 2.** Percentage of variants found in different regions of the genome. **Supplementary Figure 3.** Decontamination of the giant kelp reference genome. Blobtools results showing the distribution of GC content, coverage, and taxonomic classification. Filters selected contigs with GC content between 0.35-0.65 with coverage between 5x-300x, with the addition of 29 contigs manually checked. **Supplementary Table 1.** Summary of repetitive elements in the giant kelp genome. **Supplementary Table 2.** Summary of impact of mutation by functional class.

**Additional file 2.**

**Additional file 3.**

## Data Availability

The dataset generated and used for genome assembly during the current study is available in the NCBI repository under the accession code PRJNA926673 (Reviewer link: https://dataview.ncbi.nlm.nih.gov/object/PRJNA926673?reviewer=cr4jjkgr7lm93vc491mb4g5aro). Final genome assembly an annotation files are available at the Joint Genome Institute portal https://phycocosm.jgi.doe.gov/Macpyr2. The dataset analyzed for variant calling is available in the NCBI repository under the accession code PRJNA661280.

## References

[1] Rassweiler A, Reed DC, Harrer SL, Nelson JC (2018). Improved estimates of net primary production, growth, and standing crop of *Macrocystis pyrifera* in Southern California. Ecology.

[2] Buschmann A, Graham M, Vasquez J. Global ecology of the giant kelp Macrocystis: from ecotypes to ecosystems. In: Gibson R, Atkinson R, Gordon J, editors. Oceanography and marine biology. CRC Press; 2007. p. 39–88. (Oceanography and marine biology - an annual review; vol. 20074975). Available from: http://www.crcnetbase.com/doi/abs/10.1201/9781420050943.ch2. Cited 2023 Jan 26.

[3] Reed DC, Brzezinski MA. Kelp forests. In: The management of natural coastal carbon sinks. 2009. p. 31.

[4] Schiel DR, Foster MS. The biology and ecology of giant kelp forests. Univ of California Press; 2015.

[5] Darwin C. The voyage of the Beagle. New York : P. F. Collier & Son; 1909. Available from: http://www.biodiversitylibrary.org/bibliography/98662. Cited 2023 Jan 26.

[6] Boland W, Marner FJ, Jaenicke L, Muller DG, Folster E (1983). Comparative receptor study in gamete chemotaxis of the seaweeds Ectocarpus siliculosus and Cutleria multifida. An approach to interspecific communication of algal gametes. Eur J Biochem.

[7] Gaylord B, Reed DC, Raimondi PT, Washburn L, McLean SR (2002). A physically based model of macroalgal spore dispersal in the wave and current-dominated nearshore. Ecology.

[8] Gaylord B, Reed DC, Washburn L, Raimondi PT (2004). Physical–biological coupling in spore dispersal of kelp forest macroalgae. J Mar Syst.

[9] Gaylord B, Reed DC, Raimondi PT, Washburn L (2006). Macroalgal spore dispersal in coastal environments: mechanistic insights revealed by theory and experiment. Ecol Monogr.

[10] Reed DC (1990). The effects of variable settlement and early competition on patterns of kelp recruitment. Ecology.

[11] Reed DC, Neushul M, Ebeling AW (1991). Role of settlement density on gametophyte growth and reproduction in the kelps Pterygophora californica and Macrocystis pyrifera (phaeophyceae)1. J Phycol.

[12] Reed DC, Anderson TW, Ebeling AW, Anghera M (1997). The role of reproductive synchrony in the colonization potential of kelp. Ecology.

[13] Reed DC, Schroeter SC, Raimondi PT (2004). Spore supply and habitat availability as sources of recruitment limitation in the giant kelp Macrocystis pyrifera (phaeophyceae)1: colonization in giant kelp. J Phycol.

[14] Castorani MCN, Reed DC, Alberto F, Bell TW, Simons RD, Cavanaugh KC (2015). Connectivity structures local population dynamics: a long-term empirical test in a large metapopulation system. Ecology.

[15] Castorani MCN, Reed DC, Raimondi PT, Alberto F, Bell TW, Cavanaugh KC (1847). Fluctuations in population fecundity drive variation in demographic connectivity and metapopulation dynamics. Proc Biol Sci.

[16] The State of World Fisheries and Aquaculture 2022. FAO; 2022. Available from: http://www.fao.org/documents/card/en/c/cc0461en. Cited 2023 Jan 26.

[17] Reyes-Tisnado R, Hernández-Carmona G, Rodríguez Montesinos YE, Arvizu Higuera DL, López GF (2005). Food grade alginates extracted from the giant kelp Macrocystis pyrifera at pilot-plant scale. Rev Investig Mar.

[18] Mollah MZI, Zahid HM, Mahal Z, Faruque MRI, Khandaker MU (2021). The usages and potential uses of alginate for healthcare applications. Front Mol Biosci.

[19] Chopin T, Tacon AGJ (2021). Importance of seaweeds and extractive species in global aquaculture production. Rev Fish Sci Aquac.

[20] Camus C, Ballerino P, Delgado R, Olivera-Nappa Á, Leyton C, Buschmann AH (2016). Scaling up bioethanol production from the farmed brown macroalga *Macrocystis pyrifera* in Chile: kelp based bioethanol production. Biofuels Bioprod Biorefin.

[21] Sohn CH. The seaweed resources of Korea. In: Seaweed resources of the world. Japan International Cooperation Agency; 1998. pp. 15–33.

[22] Hwang EK, Yotsukura N, Pang SJ, Su L, Shan TF (2019). Seaweed breeding programs and progress in eastern Asian countries. Phycologia.

[23] Liu F, Sun X, Wang F, Wang W, Liang Z, Lin Z (2014). Breeding, economic traits evaluation, and commercial cultivation of a new Saccharina variety “Huangguan No. 1”. Aquacult Int.

[24] Briggs SP (1998). Plant genomics: more than food for thought. Proc Natl Acad Sci U S A.

[25] Cock JM, Sterck L, Rouzé P, Scornet D, Allen AE, Amoutzias G (2010). The Ectocarpus genome and the independent evolution of multicellularity in brown algae. Nature.

[26] Ye N, Zhang X, Miao M, Fan X, Zheng Y, Xu D (2015). Saccharina genomes provide novel insight into kelp biology. Nat Commun.

[27] Shan T, Yuan J, Su L, Li J, Leng X, Zhang Y (2020). First genome of the brown alga Undaria pinnatifida: chromosome-level assembly using PacBio and Hi-C technologies. Front Genet.

[28] Kaye AM, Wasserman WW (2021). The genome atlas: navigating a new era of reference genomes. Trends Genet.

[29] Loureiro R, Gachon CMM, Rebours C (2015). Seaweed cultivation: potential and challenges of crop domestication at an unprecedented pace. New Phytol.

[30] Robinson N, Winberg P, Kirkendale L (2013). Genetic improvement of macroalgae: status to date and needs for the future. J Appl Phycol.

[31] Konotchick T, Dupont CL, Valas RE, Badger JH, Allen AE (2013). Transcriptomic analysis of metabolic function in the giant kelp, *Macrocystis pyrifera*, across depth and season. New Phytol.

[32] Paul S, Salavarría E, García K, Reyes-Calderón A, Gil-Kodaka P, Samolski I (2022). Insight into the genome data of commercially important giant kelp Macrocystis pyrifera. Data Brief.

[33] Molano G, Diesel J, Montecinos GJ, Alberto F, Nuzhdin SV (2022). Sporophyte stage genes exhibit stronger selection than gametophyte stage genes in haplodiplontic giant kelp. Front Mar Sci.

[34] Coyer JA, Smith GJ, Andersen RA (2001). Evolution of Macrocystis spp. (phaeophyceae) as determined by ITS1 and ITS2 sequences1. J Phycol.

[35] Starko S, Soto Gomez M, Darby H, Demes KW, Kawai H, Yotsukura N (2019). A comprehensive kelp phylogeny sheds light on the evolution of an ecosystem. Mol Phylogenet Evol.

[36] Johansson ML, Alberto F, Reed DC, Raimondi PT, Coelho NC, Young MA (2015). Seascape drivers of *M**acrocystis pyrifera* population genetic structure in the northeast Pacific. Mol Ecol.

[37] Gonzalez ST, Alberto F, Molano G. Whole-genome sequencing distinguishes the two most common giant kelp ecomorphs. Evolution. 2023;77(6):1354–69. 10.1093/evolut/qpad045 36929706

[38] Goecke F, Gómez Garreta A, Martín-Martín R, Rull Lluch J, Skjermo J, Ergon Å (2022). Nuclear DNA content variation in different life cycle stages of sugar kelp, Saccharina latissima. Mar Biotechnol.

[39] Müller DG, Maier I, Marie D, Westermeier R (2016). Nuclear DNA level and life cycle of kelps: evidence for sex-specific polyteny in *Macrocystis* (Laminariales, Phaeophyceae). J Phycol.

[40] Ahmed S, Cock JM, Pessia E, Luthringer R, Cormier A, Robuchon M (2014). A haploid system of sex determination in the brown alga Ectocarpus sp. Curr Biol.

[41] Lipinska AP, Ahmed S, Peters AF, Faugeron S, Cock JM, Coelho SM (2015). Development of PCR-based markers to determine the sex of kelps. PLoS One.

[42] Graf L, Shin Y, Yang JH, Choi JW, Hwang IK, Nelson W (2021). A genome-wide investigation of the effect of farming and human-mediated introduction on the ubiquitous seaweed Undaria pinnatifida. Nat Ecol Evol.

[43] Gurevich A, Saveliev V, Vyahhi N, Tesler G (2013). QUAST: quality assessment tool for genome assemblies. Bioinformatics.

[44] Singh RP, Bijo AJ, Baghel RS, Reddy CRK, Jha B (2011). Role of bacterial isolates in enhancing the bud induction in the industrially important red alga Gracilaria dura: Gracilaria dura-bacterial interaction. FEMS Microbiol Ecol.

[45] Weinberger F. Epiphyte-host interactions: Gracilaria conferta (Rhodophyta) and associated bacteria. 1999.

[46] Phillips N, Smith CM, Morden CW (2001). An effective DNA extraction protocol for brown algae. Phycol Res.

[47] Merchant S, Wood DE, Salzberg SL (2014). Unexpected cross-species contamination in genome sequencing projects. PeerJ.

[48] Dittami SM, Corre E (2017). Detection of bacterial contaminants and hybrid sequences in the genome of the kelp Saccharina japonica using Taxoblast. PeerJ.

[49] Li H (2018). Minimap2: pairwise alignment for nucleotide sequences. Birol I, editor. Bioinformatics.

[50] Li H, Handsaker B, Wysoker A, Fennell T, Ruan J, Homer N (2009). The sequence alignment/map format and SAMtools. Bioinformatics.

[51] Koren S, Walenz BP, Berlin K, Miller JR, Bergman NH, Phillippy AM (2017). Canu: scalable and accurate long-read assembly via adaptive *k* -mer weighting and repeat separation. Genome Res.

[52] Vaser R, Sović I, Nagarajan N, Šikić M (2017). Fast and accurate de novo genome assembly from long uncorrected reads. Genome Res.

[53] Kent J. faSplit. 2022. https://github.com/ucscGenomeBrowser/kent.

[54] Buchfink B, Xie C, Huson DH (2015). Fast and sensitive protein alignment using DIAMOND. Nat Methods.

[55] UniProt Consortium (2021). UniProt: the universal protein knowledgebase in 2021. Nucleic Acids Res.

[56] Lieberman-Aiden E, van Berkum NL, Williams L, Imakaev M, Ragoczy T, Telling A (2009). Comprehensive mapping of long-range interactions reveals folding principles of the human genome. Science.

[57] Li H, Durbin R (2010). Fast and accurate long-read alignment with Burrows-Wheeler transform. Bioinformatics.

[58] Faust GG, Hall IM (2014). SAMBLASTER: fast duplicate marking and structural variant read extraction. Bioinformatics.

[59] Durand NC, Robinson JT, Shamim MS, Machol I, Mesirov JP, Lander ES (2016). Juicebox provides a visualization system for Hi-C contact maps with unlimited zoom. Cell Syst.

[60] Rao SSP, Huntley MH, Durand NC, Stamenova EK, Bochkov ID, Robinson JT (2014). A 3D map of the human genome at kilobase resolution reveals principles of chromatin looping. Cell.

[61] Bickhart DM, Rosen BD, Koren S, Sayre BL, Hastie AR, Chan S (2017). Single-molecule sequencing and chromatin conformation capture enable de novo reference assembly of the domestic goat genome. Nat Genet.

[62] Burton JN, Adey A, Patwardhan RP, Qiu R, Kitzman JO, Shendure J (2013). Chromosome-scale scaffolding of de novo genome assemblies based on chromatin interactions. Nat Biotechnol.

[63] Cobbin JC, Charon J, Harvey E, Holmes EC, Mahar JE (2021). Current challenges to virus discovery by meta-transcriptomics. Curr Opin Virol.

[64] Manni M, Berkeley MR, Seppey M, Zdobnov EM. BUSCO: assessing genomic data quality and beyond. Curr Protoc. 2021;1(12). Available from: https://onlinelibrary.wiley.com/doi/10.1002/cpz1.323. Cited 2023 Jan 26. 10.1002/cpz1.323 34936221

[65] Hanschen ER, Hovde BT, Starkenburg SR (2020). An evaluation of methodology to determine algal genome completeness. Algal Res.

[66] Alhakami H, Mirebrahim H, Lonardi S (2017). A comparative evaluation of genome assembly reconciliation tools. Genome Biol.

[67] Phillips N, Kapraun DF, Gómez Garreta A, Ribera Siguan MA, Rull JL, Salvador Soler N (2011). Estimates of nuclear DNA content in 98 species of brown algae (Phaeophyta). AoB Plants.

[68] Soler NS, Lluch JR, Garreta AG (2019). Intraindividual variation in nuclear DNA content in Durvillaea antarctica (Chamisso) Hariot, Macrocystis pyrifera (Linnaeus) C. Agardh and Lessonia spicata (Suhr) Santelices (Phaeophyceae). Cryptogam Algol.

[69] Li X, Waterman MS (2003). Estimating the repeat structure and length of DNA sequences using ℓ-tuples. Genome Res.

[70] Pflug JM, Holmes VR, Burrus C, Johnston JS, Maddison DR (2020). Measuring genome sizes using read-depth, k-mers, and flow cytometry: methodological comparisons in beetles (Coleoptera). G3.

[71] Kokot M, Dlugosz M, Deorowicz S (2017). KMC 3: counting and manipulating k-mer statistics. Bioinformatics.

[72] Grigoriev IV, Nikitin R, Haridas S, Kuo A, Ohm R, Otillar R (2014). MycoCosm portal: gearing up for 1000 fungal genomes. Nucl Acids Res.

[73] Kuo A, Bushnell B, Grigoriev IV. Fungal genomics. In: Advances in botanical research. Elsevier; 2014. p. 1–52. Available from: https://linkinghub.elsevier.com/retrieve/pii/B978012397940700001X. Cited 2023 Jan 26.

[74] Smit A, Hubley R, Green P. RepeatMasker Open-4.0. 2013. Available from: http://www.repeatmasker.org.

[75] Jurka J, Kapitonov VV, Pavlicek A, Klonowski P, Kohany O, Walichiewicz J (2005). Repbase Update, a database of eukaryotic repetitive elements. Cytogenet Genome Res.

[76] Price AL, Jones NC, Pevzner PA (2005). De novo identification of repeat families in large genomes. Bioinformatics.

[77] Salamov AA, Solovyev VV (2000). Ab initio gene finding in *Drosophila* genomic DNA. Genome Res.

[78] Ter-Hovhannisyan V, Lomsadze A, Chernoff YO, Borodovsky M (2008). Gene prediction in novel fungal genomes using an ab initio algorithm with unsupervised training. Genome Res.

[79] Birney E, Clamp M, Durbin R (2004). GeneWise and Genomewise. Genome Res.

[80] Zhou K, Salamov A, Kuo A, Aerts AL, Kong X, Grigoriev IV (2015). Alternative splicing acting as a bridge in evolution. Stem Cell Investig.

[81] Hoff KJ, Lange S, Lomsadze A, Borodovsky M, Stanke M (2016). BRAKER1: unsupervised RNA-Seq-based genome annotation with GeneMark-ET and AUGUSTUS: Table 1. Bioinformatics.

[82] Grabherr MG, Haas BJ, Yassour M, Levin JZ, Thompson DA, Amit I (2011). Full-length transcriptome assembly from RNA-Seq data without a reference genome. Nat Biotechnol.

[83] Kim D, Paggi JM, Park C, Bennett C, Salzberg SL (2019). Graph-based genome alignment and genotyping with HISAT2 and HISAT-genotype. Nat Biotechnol.

[84] Nielsen H, Engelbrecht J, Brunak S, von Heijne G (1997). Identification of prokaryotic and eukaryotic signal peptides and prediction of their cleavage sites. Protein Eng Des Sel.

[85] Melén K, Krogh A, von Heijne G (2003). Reliability measures for membrane protein topology prediction algorithms. J Mol Biol.

[86] Quevillon E, Silventoinen V, Pillai S, Harte N, Mulder N, Apweiler R (2005). InterProScan: protein domains identifier. Nucleic Acids Res.

[87] Kanehisa M (2006). From genomics to chemical genomics: new developments in KEGG. Nucleic Acids Res.

[88] Flynn JM, Hubley R, Goubert C, Rosen J, Clark AG, Feschotte C (2020). RepeatModeler2 for automated genomic discovery of transposable element families. Proc Natl Acad Sci USA.

[89] Grigoriev IV, Hayes RD, Calhoun S, Kamel B, Wang A, Ahrendt S (2021). PhycoCosm, a comparative algal genomics resource. Nucleic Acids Res.

[90] Huson DH, Scornavacca C (2012). Dendroscope 3: an interactive tool for rooted phylogenetic trees and networks. Syst Biol.

[91] Krzywinski M, Schein J, Birol İ, Connors J, Gascoyne R, Horsman D (2009). Circos: an information aesthetic for comparative genomics. Genome Res.

[92] Krueger F, James F, Ewels P, Afyounian E, Schuster-Boeckler B. FelixKrueger/TrimGalore: v0.6.7 - DOI via Zenodo. Zenodo; 2021. Available from: https://zenodo.org/record/5127899. Cited 2023 Jan 26.

[93] Danecek P, Auton A, Abecasis G, Albers CA, Banks E, DePristo MA (2011). The variant call format and VCFtools. Bioinformatics.

[94] Van der Auwera GA, Carneiro MO, Hartl C, Poplin R, del Angel G, Levy‐Moonshine A, et al. From FastQ data to high‐confidence variant calls: the genome analysis toolkit best practices pipeline. Curr Protoc Bioinformatics. 2013;43(1). Available from: https://onlinelibrary.wiley.com/doi/10.1002/0471250953.bi1110s43. Cited 2023 Jan 26. 10.1002/0471250953.bi1110s43 PMC4243306 25431634

[95] Zheng X, Levine D, Shen J, Gogarten SM, Laurie C, Weir BS (2012). A high-performance computing toolset for relatedness and principal component analysis of SNP data. Bioinformatics.

[96] Wickham H. ggplot2: elegant graphics for data analysis. 2nd ed. 2016. Cham: Springer International Publishing: Imprint: Springer; 2016. p. 1. (Use R!).

[97] Webb A, Knoblauch J, Sabankar N, Kallur AS, Hey J, Sethuraman A (2021). The pop-gen pipeline platform: a software platform for population genomic analyses. Harris K, editor. Mol Biol Evol.

[98] Browning BL, Tian X, Zhou Y, Browning SR (2021). Fast two-stage phasing of large-scale sequence data. Am J Hum Genet.

