# Supplementary material for: A scaffolded and annotated reference genome of giant kelp (Macrocystis pyrifera)
Source: BMC Genomics. 2023 Sep 13;24:543. doi: 10.1186/s12864-023-09658-x (PMC10498591; doi:10.1186/s12864-023-09658-x)
Supplement: Supplementary file 1 — Additional file 1: Supplementary Figure 1. Kmer distributions generated from corrected Pacbio reads. The genome size can be estimated by summing the total number of kmers found in the Pacbio reads and dividing by the peak of kmers (essentially the coverage for the genome). For a kmer size of k = 25, the coverage was x28 and the genome estimate was ~495 MB. For a kmer size of k = 31, the coverage was x27 and the genome estimate was ~523 MB. Supplementary Figure 2. Percentage of variants found in different regions of the genome. Supplementary Figure 3. Decontamination of the giant kelp reference genome. Blobtools results showing the distribution of GC content, coverage, and taxonomic classification. Filters selected contigs with GC content between 0.35-0.65 with coverage between 5x-300x, with the addition of 29 contigs manually checked. Supplementary Table 1. Summary of repetitive elements in the giant kelp genome. Supplementary Table 2. Summary of impact of mutation by functional class. [file 12864_2023_9658_MOESM1_ESM.docx]

**Supplementary Materials**

**1 Supplementary Figures and Tables**

**1.1 Supplementary Figures**


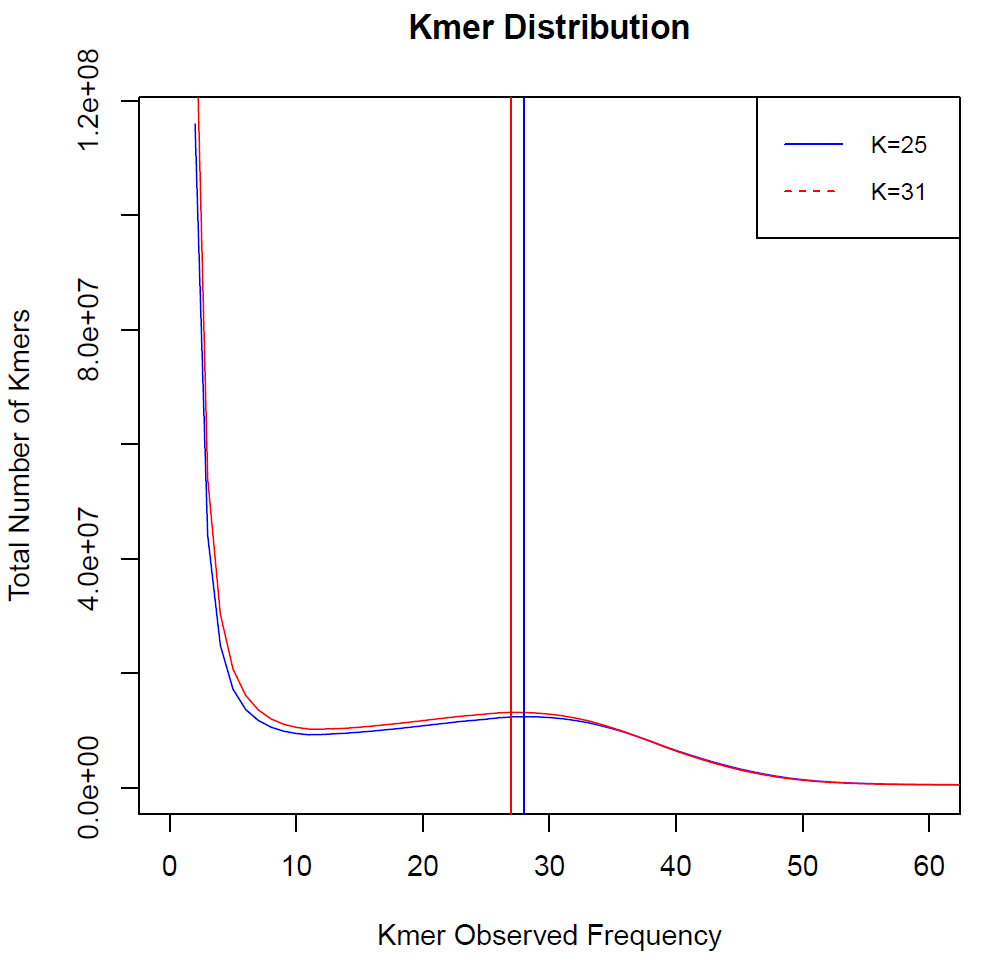


**Supplementary Figure 1.** Kmer distributions generated from corrected Pacbio reads. The genome size can be estimated by summing the total number of kmers found in the Pacbio reads and dividing by the peak of kmers (essentially the coverage for the genome). For a kmer size of k=25, the coverage was x28 and the genome estimate was ~495 MB. For a kmer size of k=31, the coverage was x27 and the genome estimate was ~523 MB.

**Supplementary Figure 2.** Percentage of variants found in different regions of the genome.

**
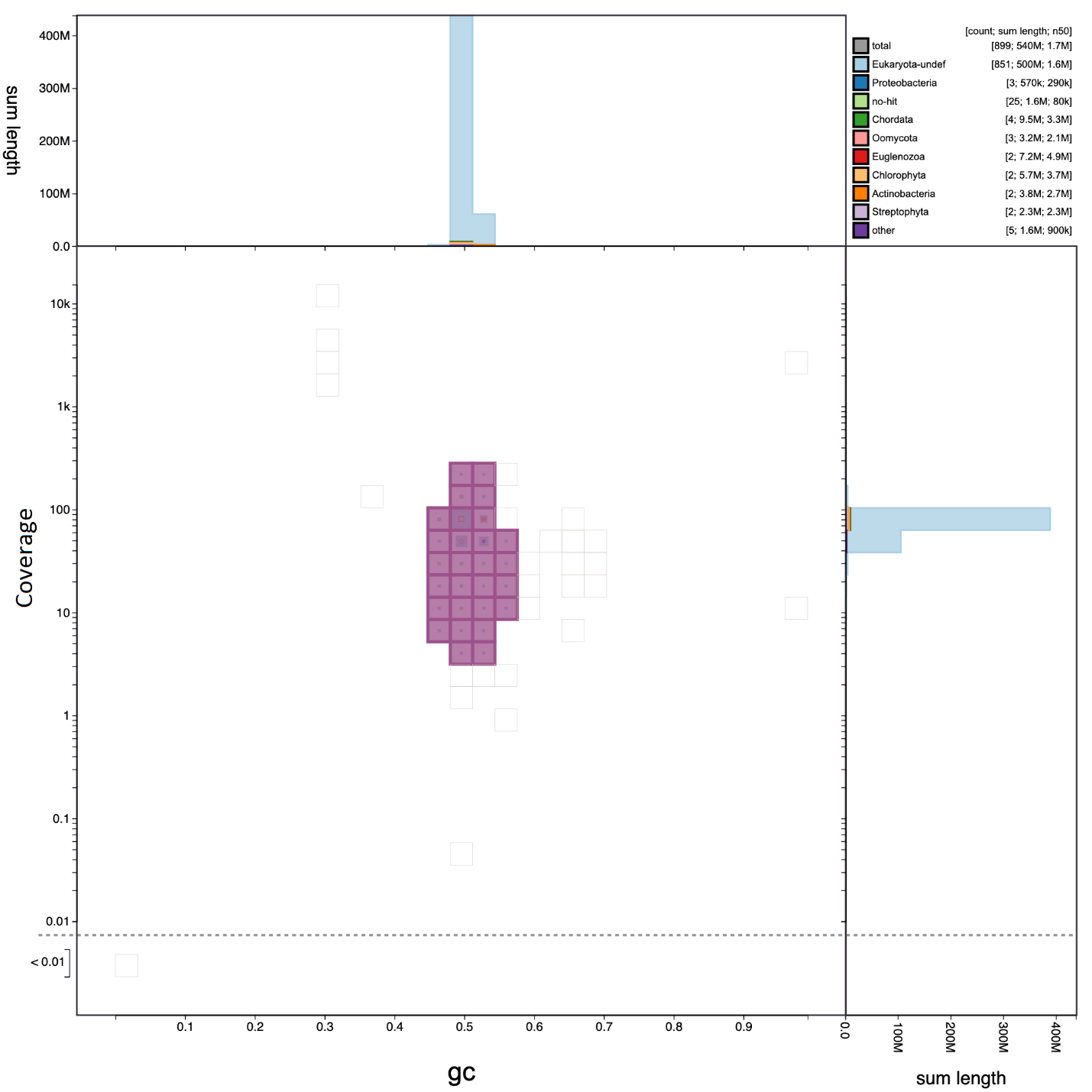
**

**Supplementary Figure 3.** Decontamination of the giant kelp reference genome. Blobtools results showing the distribution of GC content, coverage, and taxonomic classification. Filters selected contigs with GC content between 0.35-0.65 with coverage between 5x-300x, with the addition of 29 contigs manually checked.

**1.2 Supplementary Tables**

**Supplementary Table 1.** Summary of repetitive elements in the giant kelp genome.

|  | # of elements | Total length (bp) | % Of sequence |
| --- | --- | --- | --- |
| Retroelements | 221242 | 99035057 | 18.43 |
| *LINEs* | 76806 | 28515654 | 5.31 |
| *LTR* | 144436 | 70519403 | 13.12 |
| DNA transposons | 4184 | 980757 | 0.18 |
| *Hobo-Activator* | 3166 | 496193 | 0.09 |
| Rolling circles | 2176 | 1466554 | 0.27 |
| Unclassified | 869640 | 191659760 | 35.66 |
| interspersed repeats | NA | 291675574 | 54.27 |
| Small RNA | 6407 | 1165914 | 0.22 |
| Simple repeats | 244164 | 13836255 | 2.57 |

**Supplementary Table 2.** Summary of impact of mutation by functional class.

| Type of mutation | Count | Percent |
| --- | --- | --- |
| Missense | 227696 | 53.196 |
| nonsense | 3629 | 0.848 |
| silent | 196706 | 45.956 |
